# Hepatitis transactivator protein X promotes extracellular matrix modification through HIF/LOX pathway in liver cancer

**DOI:** 10.1038/s41389-018-0052-8

**Published:** 2018-05-25

**Authors:** Aki Pui-Wah Tse, Karen Man-Fong Sze, Queenie Tsung-Kwan Shea, Elley Yung-Tuen Chiu, Felice Ho-Ching Tsang, David Kung-Chun Chiu, Misty Shuo Zhang, Derek Lee, Iris Ming-Jing Xu, Cerise Yuen-Ki Chan, Hui-Yu Koh, Chun-Ming Wong, Yong-Ping Zheng, Irene Oi-Lin Ng, Carmen Chak-Lui Wong

**Affiliations:** 1 Department of Pathology, The University of Hong Kong, Hong Kong, China; 2 Department of Biomedical Engineering, The Hong Kong Polytechnic University, Hong Kong, China; 3 State Key Laboratory for Liver Research, The University of Hong Kong, Hong Kong, China

## Abstract

Hepatocellular carcinoma (HCC), accounting for 90% of primary liver cancer, is a lethal malignancy that is tightly associated with chronic hepatitis B virus (HBV) infection. HBV encodes a viral onco-protein, transactivator protein X (HBx), which interacts with proteins of hepatocytes to promote oncogenesis. Our current study focused on the interaction of HBx with a transcription factor, hypoxia-inducible factor-1α (HIF-1α), which is stabilized by low O_2_ condition (hypoxia) and is found to be frequently overexpressed in HCC intra-tumorally due to poor blood perfusion. Here, we showed that overexpression of HBx by tetracycline-inducible systems further stabilized HIF-1α under hypoxia in HBV-negative HCC cell lines. Reversely, knockdown of HBx reduced HIF-1α protein stabilization under hypoxia in HBV-positive HCC cell lines. More intriguingly, overexpression of HBx elevated the mRNA and protein expression of a family of HIF-1α target genes, the lysyl oxidase (LOX) family in HCC. The LOX family members function to cross-link collagen in the extracellular matrix (ECM) to promote cancer progression and metastasis. By analyzing the collagens under scanning electron microscope, we found that collagen fibers were significantly smaller in size when incubated with conditioned medium from HBx knockdown HCC cells as compared to control HCC cells in vitro. Transwell invasion assay further revealed that less cells were able to invade through the matrigel which was pre-treated with conditioned medium from HBx knockdown HCC cells as compared to control HCC cells. Orthotopic and subcutaneous HCC models further showed that knockdown of HBx in HCC cells reduced collagen crosslinking and stiffness in vivo and repressed HCC growth and metastasis. Taken together, our in vitro and in vivo studies showed the HBx remodeled the ECM through HIF-1α/LOX pathway to promote HCC metastasis.

## Introduction

Hepatocellular carcinoma (HCC), malignancy transformed from hepatocytes, accounts for 90% of primary liver cancer. HCC is the fifth most prevalent cancer and the third leading cause of cancer-associated death. The high mortality rate of HCC is mainly associated with late symptom presentation in patients. Most HCC patients are diagnosed at advanced stages when they have missed the opportunity for surgical treatments. Currently, the only FDA approved first-line targeted therapy for HCC, Sorafenib (tyrosine kinase inhibitor, TKI), could lengthen the survival time of patients for less than three months^1,2^. Regorafenib (TKI) and Nivolumab (immune checkpoint inhibitor targeting programmed death 1 [PD1]) were recently approved by FDA as second-line treatments^3,4^. Regorafenib could only prolong the survival of Sorafenib-resistant HCC patients for another 2–3 months^3^. Meanwhile, Nivolumab has an unprecedented response rate of 20% in HCC patients^4^. Nivolumab, through blocking inhibitory T cell receptor PD1, reactivates T cells to suppress HCC. Nivolumab highlighted the immense potential of monoclonal antibodies against other inhibitory T cell receptors, such as TIM3 and LAG3 in HCC treatment.

Among all the etiological factors of HCC, chronic hepatitis B virus (HBV) infection is particularly relevant in our population. Complete elimination of HBV is particularly challenging as HBV forms covalent closed circular DNA in the nuclei of the host (hepatocytes) where the viral DNA integrated into the host genome^5^. Therefore, HBV infection remains a major social health burden in HK. HBV is a circular and partially double-stranded 3.2 kb DNA virus, which encodes for viral polymerase, viral envelope protein, core protein, and the viral transactivator protein X (HBx) protein. HBx is the most important HBV component contributing to HCC. Overexpression of HBx enhanced proliferation of liver cells, while knockdown of HBx expression reduced tumorigenecity of HCC cells^6,7^. HBx protein markedly promoted HCC formation in transgenic mice^8^. HBx directly interacted with and inactivated tumor suppressor p53^9^. HBx also activated NFκB transcription factor pathway and growth regulatory genes including c-fos, c-jun, c-myc, and EGF^10^.

HBx has been shown to interact with an important transcription factor, hypoxia-inducible factor (HIF)^11^. HIF confers growth advantage and metastatic potential to HCC cells by driving a wide repertoire of genes that benefit tumor development^12^. Increased protein level of HIF is observed in HCC and associated with poor prognosis^13–15^. Increased expression of HIF is mainly attributed to intratumoral hypoxia, a common phenomenon in regions of rapidly growing HCC that are distant from functional blood vessels resulting in low oxygen (O_2_) supply. HIFs are composed of the O_2_-regulated HIF-1/2α subunits and the constitutively expressed HIF-1β subunit^16^. In the presence of O_2_, HIF-1/2α is hydroxylated by prolyl hydroxylases (PHDs)^16^. Hydroxylated HIF-1/2α is recognized by the von Hippel-Lindau protein (VHL), which then interacts with the Elongin C protein, thereby recruiting E3 ubiquitin-protein ligase that targets HIF-1/2α subunit for ubiquitin-mediated proteosomal degradation^16^. HIF-1/2α then dimerizes with HIF-1β, together with co-activator CREB-binding protein (CBP)/p300, bind to the promoters of its target genes encompassing hypoxia response elements (HREs) to initiate gene transcription. HIF-1/2α induces a large number of target genes that are important for different aspects of cancer progression.

Previously, we showed that HIF-1α regulates the expression of a family of secretory proteins that are extracellular matrix modifiers: lysyl oxidase (LOX), lysyl oxidase-like 2 (LOXL2), and LOXL4^17^. Collagen is the most abundant component in the extracellular matrix of the tumor microenvironment. LOX family deaminates lysines of the collagen resulting in crosslinking of collagen, and subsequently leads to tissue stiffening, an important clinicopathological feature of cirrhosis and HCC. We and others have shown that the HIF/LOX pathway played an important role in tumor microenvironment formation in breast and liver models. LOX and LOXL2 stiffened the breast and liver cancer tissues^18,^^19^. Alteration of the stiffness in the tumor microenvironment triggered the Rho/ROCK and PI3K signaling pathways, thereby promoted the invasion of cancer cells^18,^^19^. A recent study demonstrated that substrate stiffness activated epithelial-to-mesenchymal transition (EMT), enhancing migratory ability of cells^20^. Collagen crosslinking allows formation of large collagen fibers which have been shown to act as substrates and physical scaffolds for cancer cell to adhere, migrate, and invade^21–23^. Cancer cell movement on large collagen fiber has been schematically viewed as ‘‘car on a highway’’^21–23^. Chronic HBV infection is often associated with cirrhosis which is featured by extensive ECM modeling such as massive collagen accumulation^24^. Meanwhile, LOXL2 has been shown to be responsible for liver fibrosis^25^. HBx has been shown to promote HCC progression. Studies showed that HBx stabilizes HIF-1α^11,26^. Our previous study suggested that HIF-1α induced the expression of LOXL2 which crosslinked collagen in the ECM to promote local invasion of HCC cells^19^. Therefore, we propose to investigate whether HBV infection may promote tumor microenvironment formation in HCC through HBx which induces stabilization of HIF-1α, in turn transcriptionally activates LOX family to induce collagen crosslinking.

## Results

### Establishment of tetracycline-inducible stable expression of HBx cell model and HBx knockdown cell model

**Fig. 1:**
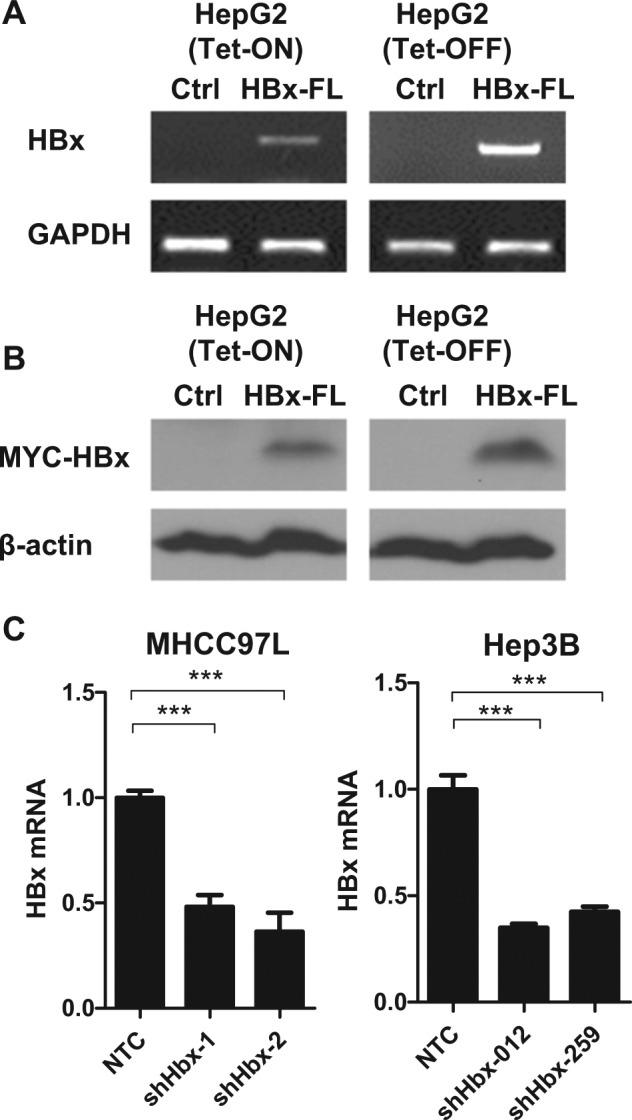
Gain-of-function and loss-of-function of HBx HCC cell models. **a** *HBx* was stably induced in tetracycline-inducible (Tet-ON or Tet-OFF) systems in HepG2. DNA from the cell lines were extracted and PCR products were run on agarose gels. *GADPH* was used as loading control. **b** myc-tagged-HBx protein was detected in tetracycline-inducible (Tet-ON or Tet-OFF) systems in HepG2 by Western blotting using MYC antibody. **c** *HBx* mRNA expressions in MHCC97L-NTC, -shHBx-01, and -shHBx-02 and Hep3B-NTC, shHBx-012, and shHBx-249 subclones were determined by qRT-PCR. *p < 0.05, **p < 0.01, ***p < 0.001, Student’s *t* test

To study the effect of HBx on HIF-1α stability, we employed gain-of-function and loss-of-function HBx models in HCC cell lines. For gain-of-function, we established tetracycline-inducible stable expression (Tet-Off and Tet-On) of HBx (NCBI: U95551) in hepatoblastoma cell line (HepG2), which do not express endogenous HBx. PCR using primers flanking *HBx* and western blotting showed that HBx was successfully expressed upon tetracycline withdrawal (Tet-OFF) or tetracycline addition (Tet-ON) (Fig. 1a, b and Supplementary Fig. 1). For loss-of-function, we used lentiviral-mediated short hairpin RNA to stably knock down HBx in HCC cell lines (MHCC97L and Hep3B), which express endogenous HBx. qRT-PCR showed that *HBx* expression was successfully reduced in HCC subclones that expressed shRNA targeting HBx as compared to the HCC subclones that expressed shRNA against non-targeting control (NTC) sequence (Fig. 1c).

### HBx stabilizes HIF-1α to activate transcription of LOX family

**Fig. 2:**
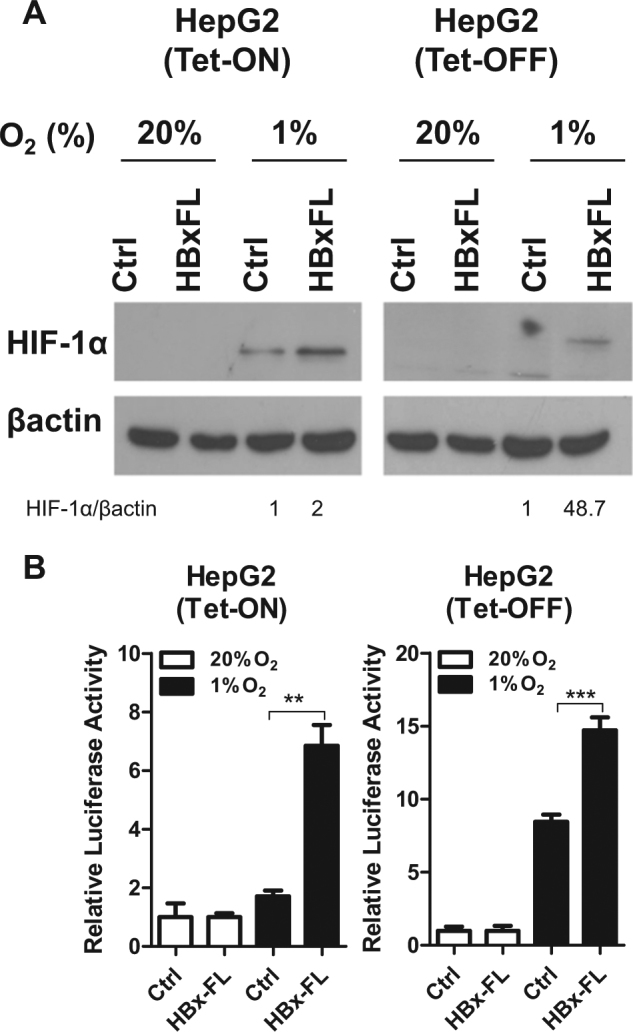
HBx stabilized HIF-1α and promoted HIF activity. **a** HIF-1α protein expression in HepG2 cells that stably express HBx that were exposed to 20 and 1% O_2_ for 4 h. **b** Dual-luciferase reporter assay was performed in HCC cell lines that were transfected with p2.1 5 × HRE Firefly luciferase and Renilla luciferase reporters. Cells were exposed to 20 and 1% O_2_ for 24 h. *p < 0.05, **p < 0.01, ***p < 0.001, Student’s *t* test

**Fig. 3:**
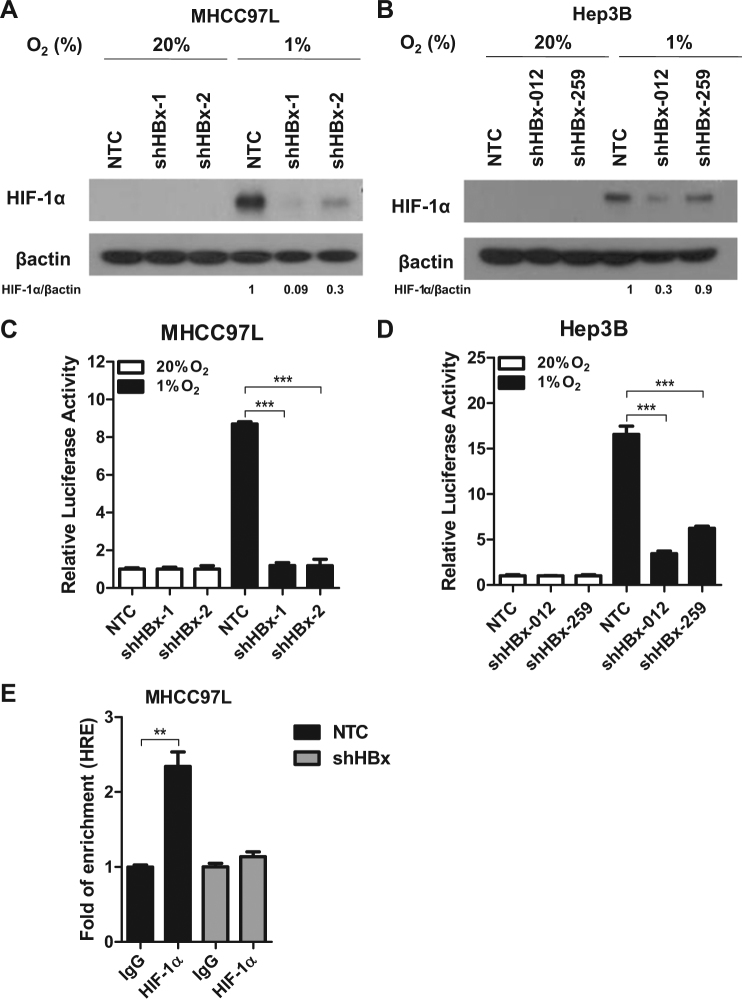
HIF-1 protein and activity were abrogated in HBx knockdown HCC cells. **a** HIF-1α protein expression in MHCC97-NTC, -shHBx-01, and -shHBx-02 stable cells. **b** HIF-1α protein expression in Hep3B-NTC, -shHBx-012, and -shHBx-259 stable cells. **c**, **d** Dual-luciferase reporter assay was performed in HBx knockdown HCC cells. MHCC97L and Hep3B subclones were transfected with p2.1 5 × HRE Firefly luciferase and Renilla luciferase reporters. Cells were exposed to 20 and 1% O_2_ for 24 h. **e** ChIP assay was performed in MHCC97L-NTC and –shHBx cells were exposed to 1% O_2_ for 24 h using HIF-1α and IgG control antibodies. RT-qPCR was performed to amplify a known DNA-binding region of HIF-1α. *p < 0.05, **p < 0.01, ***p < 0.001, Student’s *t* test

**Fig. 4:**
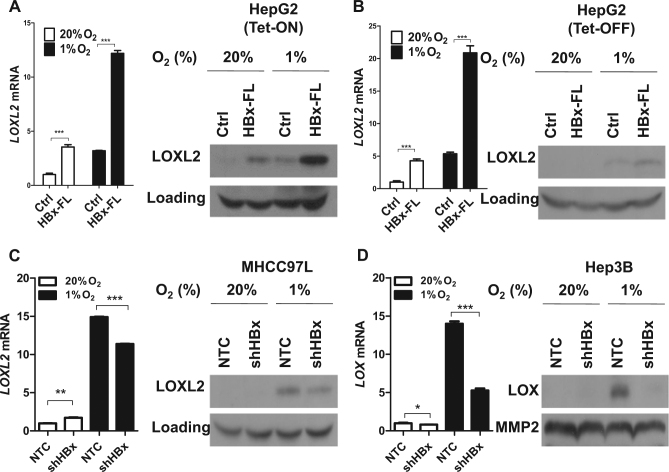
HBx induced LOXL2 mRNA and protein expressions. **a** Left: *LOXL2* mRNA expression levels in HepG2-Ctrl and –HBx-FL expressing cells that were exposed to 20 and 1% O_2_ for 24 h in the presence of tetracycline. Right: LOXL2 protein expression levels in the conditioned media of HepG2-Ctrl and –HBx-FL expressing cells that were exposed to 20 and 1% O_2_ for 48 h in the presence of tetracycline. **b** Left: *LOXL2* mRNA expression levels in HepG2-Ctrl and –HBx-FL expressing cells that were exposed to 20 and 1% O_2_ for 24 h in the absence of tetracycline. Right: LOXL2 protein expression levels in the conditioned media of HepG2-Ctrl and –HBx-FL expressing cells that were exposed to 20 and 1% O_2_ for 48 h in the absence of tetracycline. **c** Left: *LOXL2* mRNA expression levels in MHCC97L-NTC, -shHBx-01, and -shHBx-02 cells that were exposed to 20 and 1% O_2_ for 24 h. Right: LOXL2 protein expression levels in the conditioned media of MHCC97L-NTC, -shHBx-01, and -shHBx-02 cells that were exposed to 20 and 1% O_2_ for 48 h. **c** Left: *LOXL2* mRNA expression levels in HBx knockdown MHCC97L cells that were exposed to 20 and 1% O_2_ for 24 h. Right: LOXL2 protein expression levels in the conditioned media of HBx knockdown MHCC97L cells that were exposed to 20 and 1% O_2_ for 48 h. **d** Left: *LOX* mRNA expression levels in HBx knockdown Hep3B cells that were exposed to 20 and 1% O_2_ for 24 h. Right: LOX protein expression levels in the conditioned media of HBx knockdown Hep3B cells that were exposed to 20 and 1% O_2_ for 48 h. *p < 0.05, **p < 0.01, ***p < 0.001, Student’s *t* test

Interestingly, we observed a consistent increase of HIF-1α protein expression in all cell lines when HBx is expressed in both Tet-Off and Tet-On inducible systems upon hypoxia exposure (1% O_2_) (Fig. 2a). To examine whether re-expression of HBx affects HIF transcriptional activity, we performed p2.1 5 × HRE luciferase reporter assay in which five copies of the HRE were cloned in front of a promoter which drives luciferase expression^17,27^. 5 × HRE luciferase activity therefore directly indicates HIF activity. HBx expression significantly enhanced HIF activity under hypoxic condition as compared to control cells (Fig. 2b). Reversely, when we knocked down HBx in HCC cell lines that express HBx such as MHCC97L and Hep3B, we found that knockdown of HBx reduced HIF-1α protein level and HIF transcriptional activity (Fig. 3a–d). Chromatin immunoprecipitation (ChIP) assay with HIF-1α antibody was performed in MHCC97L-NTC and –shHBx cells exposed to hypoxia. RT-qPCR was performed with a pair of positive control primers flanking the DNA region where HIF-1α binds. ChIP assay confirmed that less DNA was enriched by HIF-1α antibody in the HBx knockdown HCC cells (Fig. 3e). These data confirmed that HBx stabilized the expression of HIF-1α in our cell system. Next, to study whether HBx regulates HIF-1α targets, we assessed LOX family mRNA expression in these cell lines and found that *LOXL2* mRNA was induced by hypoxia and was further upregulated by HBx expression in HepG2 (Fig. 4a, b). To confirm whether HBx also induced the secretory form of LOXL2, we collected the conditioned medium from our HBx-expressing subclones that were exposed to normoxia and hypoxia. We confirmed that HBx increased LOXL2 protein expression in hypoxia in HepG2 cells. The other way around, LOXL2 expression was reduced when we knocked down HBx in MHCC97L cells (Fig. 4c). Unlike MHCC97L, which expresses high level of LOXL2, Hep3B expresses low level of LOXL2 expression but high level of LOX. Interestingly, we found that knockdown of HBx in Hep3B also reduced LOX expression in the conditioned medium (Fig. 4d). These data confirmed that HBx/HIF-1α affects LOX family in HCC. Similar to our previous report showing different breast cancer cell lines express different LOX family members^17^, LOX and LOXL2 are distinctly expressed in Hep3B and MHCC97L, respectively.

### HBx remodels collagen and promotes cancer cell invasion

**Fig. 5:**
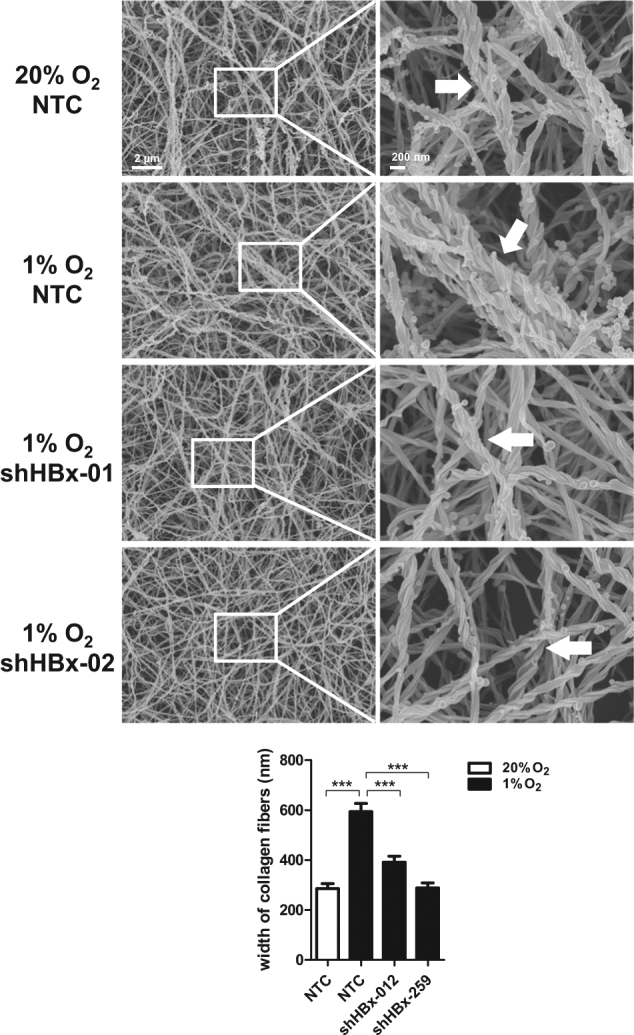
HBx induced collagen crosslinking. Top: Images of collagen I that were incubated with conditioned media from MHCC97L-NTC, -shHBx-01, and shHBx-02 cells that were exposed to 20 and 1% O_2_ for 48 h under scanning electron microscope (SEM). Bottom: Width of collagen fibers were quantitated in more than 15 microscopic fields. *p < 0.05, **p < 0.01, ***p < 0.001, Student’s *t* test

**Fig. 6:**
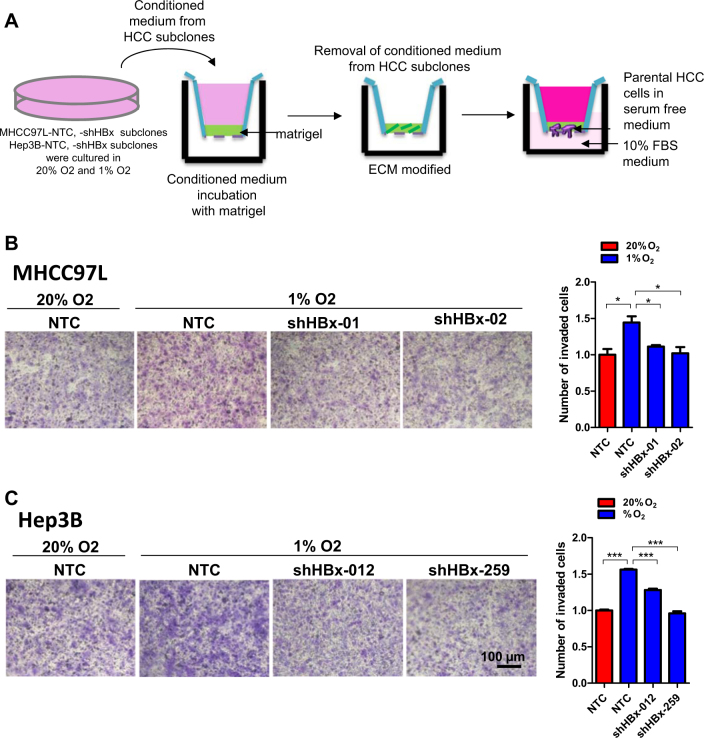
HBx promoted HCC cell invasion through ECM modification. **a** MHCC97L and Hep3B HBx knockdown HCC cells and their control cells were exposed to 20 and 1% O_2_ for 48 h. Conditioned media were collected and incubated with matrigel that was coated on the Transwell chambers. Conditioned media were removed. Parental HCC cells in serum free conditioned media were seeded onto modified ECM coated Transwell chambers and allowed to invade for 24 h. **b**, **c** Left: Parental HCC cells invaded through the matrigel-coated Transwells that were modified with conditioned media from the indicated HCC subclones. Right: number of invadesd cells were counted in at least three random fields. *p < 0.05, **p < 0.01, ***p < 0.001, Student’s *t* test

As the major function of LOX and LOXL2 is to cross-link collagen into collagen fibers, we next investigated whether HBx would affect collagen structures. We exposed MHCC97L-NTC and –shHBx cells under normoxic and hypoxic conditions and collected their conditioned medium. We then incubated the collagen-coated glass cover slips with the conditioned medium for 24 h and imaged the collagen by scanning electron microscopy (SEM). As we showed that HBx would stabilize HIF-1α and subsequently increase LOX family expressions, we hypothesize that knockdown of HBx would reduce collagen crosslinking. Strikingly, we found that hypoxia in HCC cells could markedly increase the size or thickness of the collagen fibers in the ECM (Fig. 5). This hypoxia-induced effect could be abolished in the HBx knockdown HCC cells, providing further functional consequences of the HBx/HIF-1α pathway (Fig. 5). Studies suggested that collagen crosslinking could provide invasive tracks for cancer cells to enhance their invasive abilities^28^. Changes of tension in the microenvironment would also activate cytoskeletal network^19^. To study whether HBx/HIF-1 promotes cancer cell invasion through remodeling the ECM, we treated matrigel-coated Transwells with the conditioned media collected from MHCC97L-NTC and –shHBx cells that were exposed to normoxic and hypoxic conditions (Fig. 6a). Then, we removed the conditioned medium and seeded parental cancer cells to evaluate the invasive capability through the matrigel that was pre-modified by different conditioned media (Fig. 6a). We saw that conditioned media from hypoxic HCC cells were able to increase cell invasion (Fig. 6b and Supplementary Fig. 2). This effect was abolished in the matrigel that was pre-modified by conditioned media from HBx knockdown HCC cells (Fig. 6b and Supplementary Fig. 2). The same phenomenon could be observed in Hep3B cell lines which express LOX instead of LOXL2, suggesting that the crosslinking effect is dependent on the LOX family in different HCC cell lines (Fig. 6c).

### HBx promotes HCC growth and metastasis and remodels collagen in the ECM

**Fig. 7:**
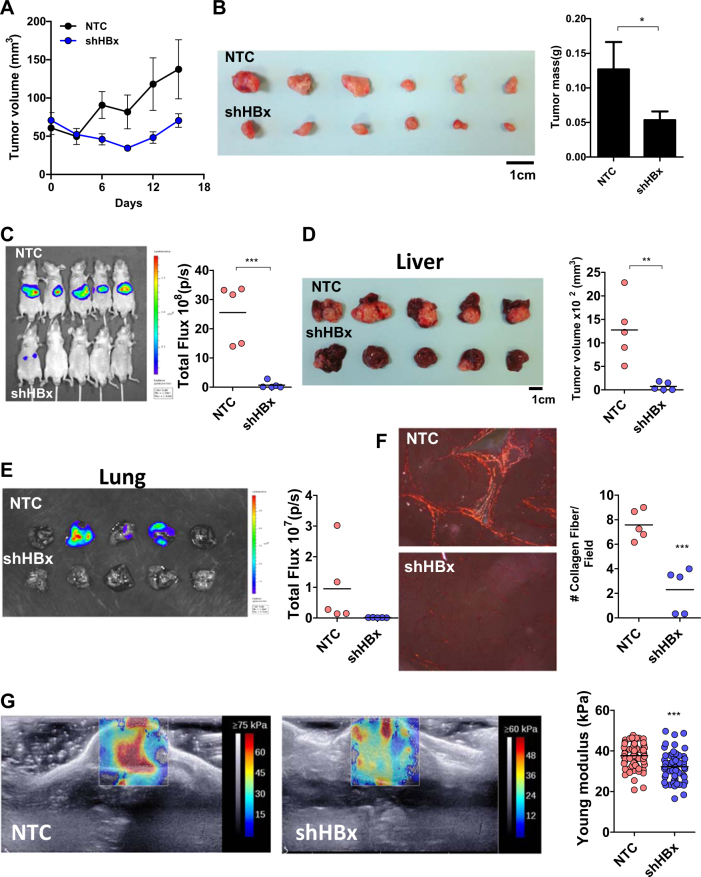
HBx promoted HCC growth and metastasis. **a** MHCC97L-NTC and –shHBx cells were inoculated into NUDE mice subcutaneously and tumor growth was monitored by caliper measurement. **b** Subcutaneous tumors were harvested and tumors were weighed. **c** Luciferase-labeled MHCC97L-NTC and –shHBx cells were inoculated into NUDE mice orthotopically. Bioluminescent images and signals were captured and quantified, respectively, in mice that were injected with luciferin. **d** Left: Images of orthotopic tumors derived from MHCC97L-NTC and –shHBx cells. Right: Tumor size was measured by caliper. **e** Bioluminiscence from lung tissues obtained from mice with orthotopic tumors derived from luciferase-labeled MHCC97L-NTC and –shHBx cells. **f** Primary orthotopic tumors were stained with picro-sirious red and analyzed under polarized light. Left: Images of crosslinked collagens of the tumors. Right: numbers of collagen fibers per field were counted in at least 10 random fields per animal at ×4 magnification. **g** Shear wave elastography imaging of subcutaneous tumors derived from MHCC97L-NTC and –shHBx cells. The mean elasticity values reflecting stiffness of the tumors were calculated. *p < 0.05, **p < 0.01, ***p < 0.001, Student’s *t* test

To confirm that HBx is affecting HCC growth, we subcutaneously inoculated MHCC97L-NTC and –shHBx cells into NUDE mice. Knockdown of HBx profoundly retarded HCC growth rate and reduced tumor size (Fig. 7a, b). We further asked whether HBx affects HCC metastasis. To more closely imitate the microenvironment of HCC, we orthotopically injected luciferase-labeled MHCC97L-NTC and –shHBx cells into the left lobes of the livers of NUDE mice (Fig. 7c). Six weeks post-implantation, mice were subjected to bioluminescent imaging. Knockdown of HBx drastically reduced primary growth in the orthotopic HCC model (Fig. 7c, d). More excitingly, knockdown of HBx also repressed metastasis of HCC cells to lung tissues as shown by ex vivo bioluminescent imaging (Fig. 7e). We asked whether this is related to collagen remodeling within the primary tumors that facilitated the invasion of HCC cells into the stroma. We performed picro-sirius red staining which specifically lights up only the crosslinked collagen fibers in polarized light on the primary tumors derived from MHCC97L-NTC and –shHBx cells (Fig. 7f). Strikingly, we saw a reduction of collagen fibers in the tumors derived from HBx knockdown HCC cells as compared to the control HCC cells, furthering confirming that HBx is influencing the collagen in the ECM of the tumor stroma (Fig. 7f). Crosslinking of collagen is associated with increased tumor stiffness. To establish tumors for shear wave elastography imaging for stiffness measurement, we subcutaneously injected MHCC97L-NTC and –shHBx cells in NUDE mice. Six weeks after injection, the mice were subjected to imaging. Tumors from HBx knockdown HCC cells were significantly softer than tumors from control clones (Fig. 7g), further confirming that the level of crosslinked collagen was positively associated with tumor stiffness. These data confirm the functional roles of HBx in HIF-1α/LOX in ECM modification and HCC progression.

## Discussion

### ECM modification during hepatocarcinogenesis

The most direct pathological consequence of collagen crosslinking is fibrosis. Fibrosis resembles the process of scarring in which activated fibroblasts deposit collagen. Fibroblasts are activated by immune cells during inflammation. HCC is usually preceded by hepatitis which triggers immune responses causing fibrosis. HCC aggressiveness is closely associated with fibrosis extensiveness^29^. More than 90% of HCC patients have cirrhotic liver disease background^30^ and 80% of HCC patients with hepatitis disease background suffer from cirrhosis^31^. Intriguingly, HCC incidence is also related to cirrhosis in alcoholic steatohepatitis and NASH-associated HCC^32,33^. Our study stems from HBV-associated HCC. Alcoholic steatohepatitis and NASH-associated fibrosis and HCC are apparently not a result of HBx stabilization of HIF-1α. Therefore, how different etiological factors contribute to fibrosis and HCC represents an exciting research area to be investigated. Studies have suggested that LOX family was expressed in fibrotic liver diseases^25^. Monoclonal antibody against LOXL2 was able to alleviate liver fibrosis^25^. It was shown that collagen deposition did not occur around hepatocytes but only restricted to fibrotic tissues^34^. Therefore, it is possible that fibroblasts are involved in collagen crosslinking in the initial stage of hepatocarcinogenesis while HCC cells and activated fibroblasts act together to further remodel the ECM as HCC develops.

### ECM regulation network

Our previous study demonstrated that *LOXL2* is also a transcriptional target of SMAD which is stimulated by TGFβ, an inflammatory factor^19^. We also showed that LOXL2 was transcriptionally and translationally repressed by two microRNAs, miR-26 and miR-29, which are often downregulated in HCC^19^. These findings suggested that multiple pathways converged to regulate ECM components in HCC^19^. Furthermore, apart from LOX family, another family of enzymes, transglutaminases (TGMs) could also cross-link collagen. Transglutaminase 2 (TGM2) is an abundant protein found in the liver but is normally located intracellularly in an inactive state. TGM secretion is tightly regulated and could be induced during cellular damages particularly under high calcium condition. Extracellular TGM2-associated ECM crosslinking is involved in the development of fibrotic disease found in kidneys, livers, and lungs and also tumor^35–37^. Interestingly, serum level of TGM2 was shown to be upregulated in HCC patients^38^. Moreover, TGM2 expression was elevated in HBV-positive HCC tissues and cell lines as compared to the HBV-negative controls^39^. TGM2 inhibition in HCC cell lines suppressed HCC cell invasion^39^. Although this study did not show whether HBV-induced TGM2 expression is caused by HBx, these findings suggested that HBV, in addition to HBx/HIF-1/LOX pathway, might remodel the ECM through other mechanisms. In addition to collagen, the ECM is composed of structural proteins such as elastin, protein-polysachharide complexes, such as proteoglycans, and adhesive glycoproteins fibronectin and laminin to attach cells to matrix. However, how other ECM components contribute to HCC development remains largely elusive. Our current study mainly focuses on collagen. As LOX family could cross-link elastin, the impact of HBx/HIF-1 on elastin modification merits further exploration.

### HBx might regulate other HIF-1α-mediated functions

HIF-1 is a multi-functional transcription factor as it transactivates genes of diverse functions. Apart from LOX family, it is very likely that HBx might induce other HIF targets. Therefore, our study hinted that HBx might also play important roles in multiple hallmarks of cancer through HIF-1 stabilization. HIF turns on genes including those that regulate cancer metabolism (*GLUT-1*, *PDK*, *MCT-4*, and *LDHA*), anigiogenesis (*VEGF*, *PDGF*), epithelial-mesenchymal transition (*SNAIL*, *ZEB*), and metastasis (*PLOD2*, *LOXs*, *MMPs*, *ANGPTL4*, and *L1CAM*)^12^. It will be exciting to explore whether HBx could reprogram the metabolic machinery, promote growth of blood vessels, and promote various steps in metastasis in HCC. A study showed that HBx could stimulate VEGF production in HCC cells and is associated with hypoxia-induced angiogenesis^40^. Another study showed that HBx could induce SNAIL protein^41^. These independent studies lent further support so show that HBx might be the central regulator to turn on HIF-1 target genes and their associated functions.

Our current study demonstrated that inducible expression of HBx in HBV-negative HCC cells facilitated the stabilization of HIF-1 to transactivate LOXL2, which crosslinked collagen in the ECM to promote HCC invasion. Reversely, knockdown of HBx in HBV-positive HCC cells reduced HIF-1 stabilization and *LOXL2* or *LOX* transcription to reduce crosslinking of collagen in the ECM to reduce HCC invasion. Given that ECM modification could be modulated by various factors and HCC patients have different disease backgrounds, it remains a challenge to study the causal relationship between HBx expression, HIF-1α, LOXL2, and collagen crosslinking in clinical specimens. Transgenic HBx mice have no prior cirrhotic background represent valuable experimental model to directly study the casual effect of HBx on collagen crosslinking and hepatocarcinogenesis in the future.

## Materials and methods

### Cell lines

Hepatoblastoma (HepG2) and HCC (Hep3B) cell lines were purchased from ATCC. HCC cell lines MHCC97L was from Fudan University (Dr. Z. Y. Tang)^42^. The cell lines were stably expressing HBx (NCBI: U95551) by the Tet-ON and Tet-OFF systems. To generate inducible stable HBx cells, HepG2 cells were first transfected with pLVX Tet-OFF or pLVX Tet-ON advanced vector (Clontec Laboratories) based on the manufacturer’s instructions. tTA(Tet-OFF/ON)-expressing cells were selected with G418 at 1 mg/mL for 14 days. Next, lentivirus containing full-length HBx in Myc/pLVX-Tight puro vector was infected into HepG2 cells and selected with 1 µg/mL puromycin for seven days. The expression of HBx is confirmed by polymerase chain reaction (PCR) with primers targeting HBx (primer sequences provided in Table 1) followed by DNA agarose gel electrophoresis. Cells were cultured in a humidified CO_2_ incubator under normoxic condition (20% O_2_) and hypoxic condition (1% O_2_). To generate HBx stable knockdown HCC cells, lentiviral-mediated short hairpin approach was employed, as described^17^. In brief, pLKO constructs encompassing shRNA sequences targeting HBx or non-target control (NTC) were co-transfected with lenti-viral packaging mix plasmids (System Biosciences) to 293FT cells. Viral particles were collected and infected into MHCC97L or Hep3B cells in the presence of polybrene. MHCC97L and Hep3B stable cells were selected by puromycin. Knockdown sequences of HBx were provided in Table 2.

**Table 1:** Primer Sequence

Primer	Sequence
HBx-forward	TCCTTTGTTTACGTCCCGTC
HBx-reverse	GCAGATGAGAAGGCACAGAC
LOXL2-forward	GGAAAGCGTACAAGCCAGAG
LOXL2-reverse	GCACTGGATCTCGTTGAGGT
18S-forward	GAGGATGAGGTGGAACGTGT
18S-reverse	AGAAGTGACGCAGCCCTCTA
HPRT-forward	CTTTGCTGACCTGCTGGATT
HPRT-reverse	CTGCATTGTTTTGCCAGTGT

**Table 2:** shRNA Sequences

shRNA	Sequence
shHBx-01-F	CCGGGGAGGCTGTAGGCATAAATTGCTCGAGCAATTTATGCCTACAGCCTCCTTTTTG
shHBx-01-R	AATTCAAAAAGGAGGCTGTAGGCATAAATTGCTCGAGCAATTTATGCCTACAGCCTCC
shHBx-02-F	CCGGGCCCAAGGTCTTACATAAGAGCTCGAGCTCTTATGTAAGACCTTGGGCTTTTTG
shHBx-02-R	AATTCAAAAAGCCCAAGGTCTTACATAAGAGCTCGAGCTCTTATGTAAGACCTTGGGC
shHBx-012-F	CCGGGCTGTACTGCCAACTGGATCCCTCGAGGGATCCAGTTGGCAGTACAGCTTTTTG
shHBx-012-R	AATTCAAAAAGCTGTACTGCCAACTGGATCCCTCGAGGGATCCAGTTGGCAGTACAGC
shHBx-259-F	CCGGCAGAGCCTGCCCAAGGTCTTACTCGAGTAAGACCTTGGGCAGGCTCTGTTTTTG
shHBx-259-R	AATTCAAAAACAGAGCCTGCCCAAGGTCTTACTCGAGTAAGACCTTGGGCAGGCTCTG

### Western blotting

For HIF-1α protein extraction, cells were scraped off in ice cold PBS. The cells were centrifuged for 5 min at 4 °C. Cell pellets were lysed in ice cold RIPA buffer for 15 min on ice and were centrifuged at 12,000× *g* for 15 min. Supernatants were collected for western blotting of HIF-1α (Western blotting and ChIP from Cell Signaling and Abcam, respectively) and actin (Sigma). To collect LOX and LOXL2 proteins in the conditioned media, 1 × 10^6^ cells were seeded in normoxic and hypoxic conditions for 48 h in serum free media. Conditioned media were concentrated 100 × by centrifugal units (Millipore) and subjected for Western blotting with the following antibodies: LOX (Abcam), LOXL2 (Abcam), MMP2 (Abcam), and MYC (Santa Cruz).

### RNA extratction and qRT-PCR

RNA was extracted by TRIzol (Sigma). Reverse transcription was carried by GeneAmp® Gold RNA PCR Reagent Kit (Thermo). qRT-PCR was performed with 1 × SYBR® Green Reagents (Life Technologies) and the primers amplifying *LOXL2*, *18* *S*, and *HPRT* (sequences provided in Table 2). C_t_ is the cycle number at which the fluorescence signal passes through the threshold. ΔC_t_ is calculated by subtracting the C_t_ of target gene (*LOXL2*) with the C_t_ of reference gene (HPRT and 18 S), and ΔΔC_t_ is calculated by subtracting the ΔC_t_ of treated cells with the ΔC_t_ of control cells.

### p2.1 5xHRE luciferase reporter assay

Different HCC stable cells were co-transfected with p2.1 5 × HRE Firefly luciferase reporter and Renilla luciferase reporter at 200:1 ratio by lipofectamine 2000 reagents, as we previously described^17^. After one night, cells were exposed to normoxia and hypoxia for 24 h. Firefly and Renilla luciferase activities were measured by Dual-Luciferase ® Reporter Assay System (Promega) following manufacturer’s protocol.

### Picro-sirius red staining

To stain the slides with picro-sirius red, we first performed dewaxing and rehydration steps with gradient of ethanol. Slides were immersed in 1 g/L of picro-sirius red stain (saturated aqueous solution of picric acid) (Sigma) for at least 60 min to obtain a near-equilibrium staining. The slides were washed in two changes of acidified water. The stained tissue sections were examined under polarized light microscope.

### Chromatin immunoprecipitation (ChIP) assay

MHCC97L-NTC and –shHBx cells exposed to 1% O_2_ for 24 h were fixed with 4% formalin and sonicated. HIF-1α (Abcam) and IgG control (Santa Cruz) antibodies were added to the pre-blocked DNA overnight followed by addition of Protein A Agarose (Millipore). DNA-Antibody-Protein A bead mixture was washed sequentially by low salt, high salt, LiCl salt, and TE washing buffers. DNA was eluted, de-crosslinked, and then purified by phenol chloroform, as described^43^. RT-qPCR was performed with positive control primers flanking hypoxia response element (HRE) of a HIF-1 target gene.

### Imaging of collagen fibers by scanning electron microscopy

A volume of 3 mg/mL rat tail collagen I (Thermo Fisher) was coated onto coverslips in 4 °C. Conditioned media from MHCC97L-NTC and –shHBx stable cells exposed to hypoxia and normoxia for 48 h under serum free condition were collected. Conditioned media were used to incubate with the collagen I-coated coverslips for 16 h at 37 °C. Collagen I was fixed with 100% ethanol followed by critical point dry and mounted on silver paste. Images were scanned and captured by LEO 1530 FEG Scanning Electron Microscope.

### Invasion assay

Transwell ® chambers (Corning) were coated with Matrigel (BD Biosciences). Conditioned media from MHCC97L or Hep3B-NTC and –shHBx stable cells exposed to normoxia and hypoxia for 48 h in serum free condition were used to treat matrigel-coated surface of Transwell ® chambers for 16 h at 37 °C. After conditioned media were removed, 4 × 10^5^ parental cancer cells were seeded onto the Transwell and allowed to invade for 24 h. Cells invaded through the ECM layer to the lower surface of the membrane were fixed with methanol and stained with crystal violet. Photographs of three randomly selected fields of the fixed cells were captured and the cells were manually counted. Number of invaded cells was also digitally evaluated by measuring the stained area with Image J. Relative positive area equals the stained area (purple)/ total area.

### Animal experiments

For subcutaneous injection, 1 × 10^6^ MHCC97L-NTC and –shHBx stable cells were injected into the flanks of BALB/C nude mice. Tumor growth was monitored by caliper measurement. For orthotopic implantation, 1 × 10^6^ luciferase-labeled MHCC97L-NTC and -shHBx cells inoculated in Matrigel (BD Biosciences) were injected into the left lobes of the livers of BALB/C nude mice. After 6 weeks, the animals received 100 mg/kg D-luciferin and bioluminescence was detected by Perkin Elmer IVIS Sepctrum. The excised lungs were harvested for ex vivo biolumiscent imaging. All animal experiments were performed according to the Animals (Control of Experiments) Ordinance (Hong Kong) and the Institute’s guidance on animal experimentation.

### Stiffness measurement

BALB/C nude mice were subcutaneously injected with 1 × 10^6^ MHCC97L-NTC and -shHBx cells. After six weeks of injection, the mice were anaesthetized using ketamine and xylazine before imaging. Shear wave elastography imaging was performed using AixPlorer Scanner (Supersonic, Paris, France) incorporating a SuperLinear™22-7lab linear transducer. Five images were taken on each tumor and the mean elasticity values were calculated within a manually selected region-of-interest (ROI) of the tumor volume. Two different types of ROI were selected in each image frame, which were the areas within the inner and outer boundaries of the tumor volume, respectively.

## Electronic supplementary material

HBx inducible system

HBx promoted HCC cell invasion through ECM modification (Independent trial from Figure 6)

Supplementary Figure Legend
