# Supplementary figures and images for: Hepatitis transactivator protein X promotes extracellular matrix modification through HIF/LOX pathway in liver cancer
Source: Oncogenesis. 2018 May 25;7(5):44. doi: 10.1038/s41389-018-0052-8 (PMC5968027; doi:10.1038/s41389-018-0052-8)

Supplementary Fig 1

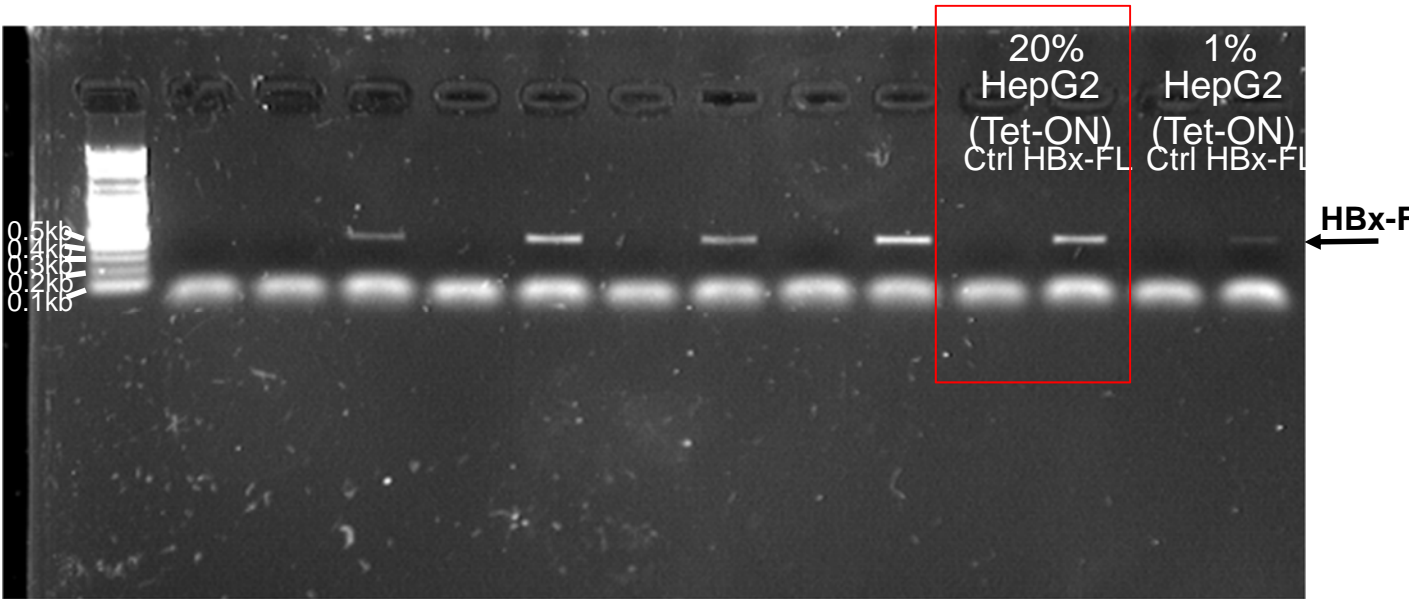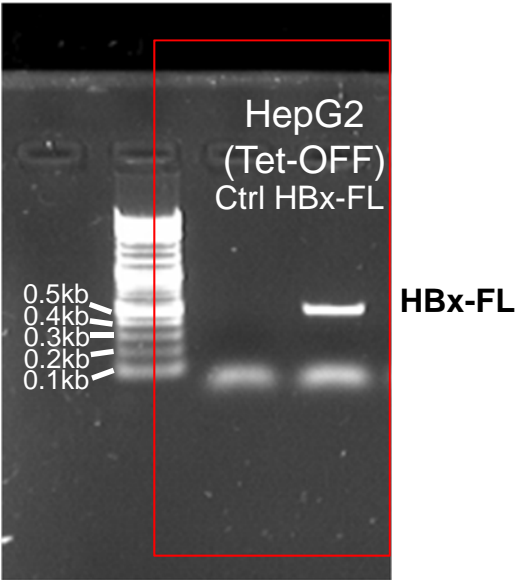

Supplement: Supplementary file 1 — HBx inducible system [file 41389_2018_52_MOESM1_ESM.pdf]

Supplementary Fig 2

MHCC97L

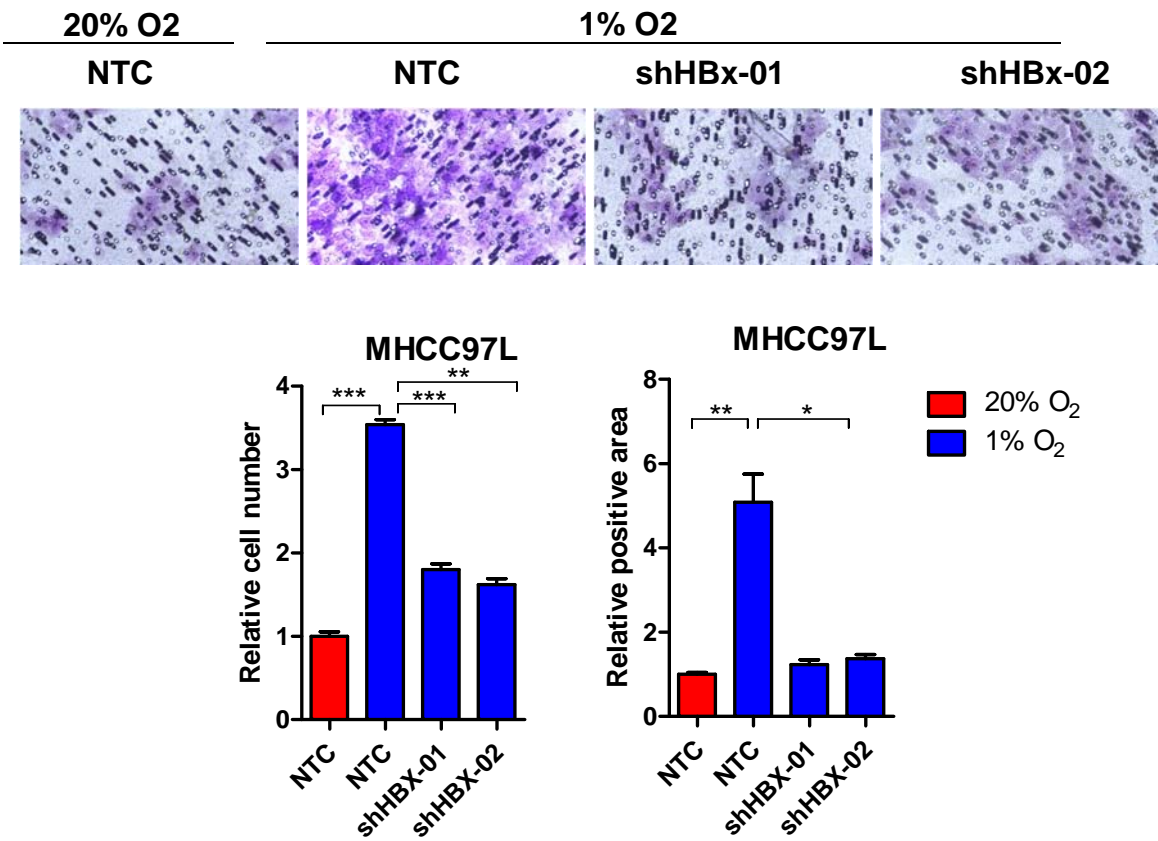

Hep3B

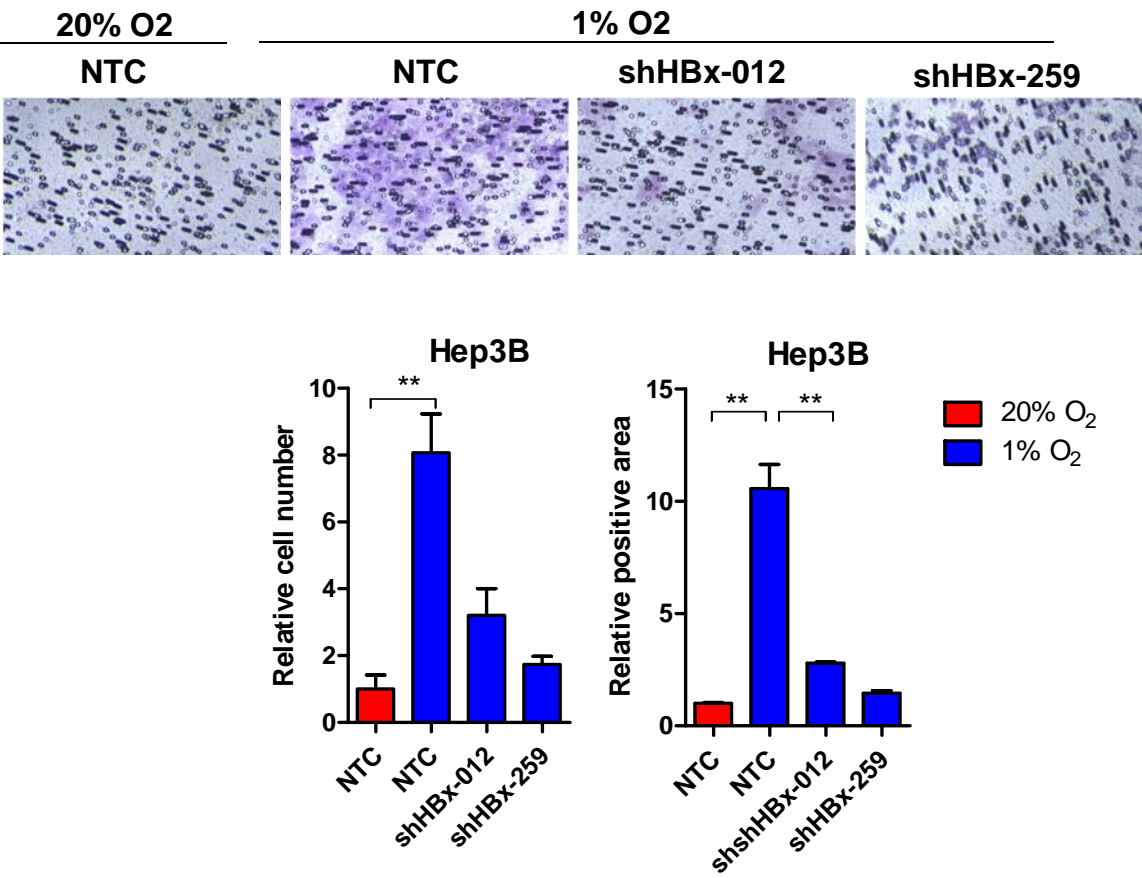

Supplement: Supplementary file 2 — HBx promoted HCC cell invasion through ECM modification (Independent trial from Figure 6) [file 41389_2018_52_MOESM2_ESM.pdf]
