# Supplementary material for: Hepatitis transactivator protein X promotes extracellular matrix modification through HIF/LOX pathway in liver cancer
Source: Oncogenesis. 2018 May 25;7(5):44. doi: 10.1038/s41389-018-0052-8 (PMC5968027; doi:10.1038/s41389-018-0052-8)
Supplement: Supplementary file 3 — Supplementary Figure Legend [file 41389_2018_52_MOESM3_ESM.docx]

**Supplementary Figure 1. HBx inducible system**. Uncropped gel photos from Figure 1 showing the HBx amplicons in the HBx inducible system.

**Supplementary Figure 2. HBx promoted HCC cell invasion through ECM modification (Independent trial from Figure 6)**. (A) MHCC97L and (B) Hep3B HBx knockdown HCC cells and their control cells were exposed to 20% and 1% O_2_ for 48 hours. Conditioned media were collected and incubated with matrigel that was coated on the Transwell chambers. Conditioned media were removed. Parental cancer cells in serum free conditioned media were seeded onto modified ECM coated Transwell chambers and allowed to invade for 24 hours. Parental cancer cells invaded through the matrigel-coated Transwells that were modified with conditioned media from the indicated HCC subclones. Intensity of invaded cells were quantitated by Image J (area with invaded cells (purple)/ total area). Numbers of invaded cells were counted in at least 3 random fields. *p<0.05, **P<0.01, ***P<0.001, Student’s t test.
